# Degradation of 3D-Printed Porous Polylactic Acid Scaffolds Under Mechanical Stimulus

**DOI:** 10.3389/fbioe.2021.691834

**Published:** 2021-10-26

**Authors:** Heming Chen, Quan Shi, Hengtao Shui, Peng Wang, Qiang Chen, Zhiyong Li

**Affiliations:** ^1^ School of Biological Science and Medical Engineering, Southeast University, Nanjing, China; ^2^ State Key Laboratory of Pharmaceutical Biotechnology, Department of Sports Medicine and Adult Reconstructive Surgery, Nanjing Drum Tower Hospital, The Affiliated Hospital of Nanjing University Medical School, Nanjing, China; ^3^ School of Mechanical, Medical and Process Engineering, Queensland University of Technology, Brisbane, QLD, Australia

**Keywords:** porous PLA scaffold, mechanical stimulus, degradation, *in vitro* experiment, numerical simulation

## Abstract

Polylactic acid (PLA) is a biodegradable polymer commonly used as a scaffold material to repair tissue defects, and its degradation is associated with mechanical stimulus. In this study, the effect of mechanical stimulus on the degradation of 3D-printed PLA scaffolds was investigated by *in vitro* experiments and an author-developed numerical model. Forty-five samples with porosity 64.8% were printed to carry out the degradation experiment within 90 days. Statistical analyses of the mass, volume fraction, Young’s modulus, and number average molecular weight were made, and the *in vitro* experiments were further used to verify the proposed numerical model of the scaffold degradation. The results indicated that the mechanical stimulus accelerated the degradation of the PLA scaffold, and the higher mechanical stimulus led to a faster degradation of the scaffolds at the late stage of the degradation process. In addition, the Young’s modulus and the normalized number average molecular weight of the PLA scaffolds between the experiments and the numerical simulations were comparable, especially for the number average molecular weight. The present study could be helpful in the design of the biodegradable PLA scaffolds.

## Introduction

Bone tissue engineering (BTE) offers a promising strategy of healing bone defects to restore their functions by utilizing the body’s natural biological response to tissue damage in conjunction with engineering principles (Amini et al., 2012). Biodegradable scaffolds are generally considered as attractive elements to provide temporary mechanical and biological supports which can facilitate regulating cell behaviors to conduct the defected bone repairment (Hutmacher, 2000). It is well-accepted that during the bone repair process, the ideal scaffolds should have a matchable degradation rate to the bone formation rate (Cao and Kuboyama, 2010; Chen et al., 2014). Meanwhile, the ideal scaffolds should provide suitable mechanical support and ultimately degrade to non-toxic products (Hutmacher et al., 1996). Therefore, much attention has been given to biodegradable synthetic aliphatic polyesters (Rezwan et al., 2006), which has shown promise in the BTE field owing to their great biocompatibility and biodegradation (Chu et al., 1999).

Polylactic acid (PLA) is one of the most widely used polyesters in BTE for its biodegradation, mechanical properties, and easy-processing advantage. The PLA biodegradation rate is affected by many factors, including morphology, molecular weight, crystallinity, and environments (e.g., external mechanical stimuli, pH value, and temperature) (Reed and Gilding, 1981; Vert, 2005; Fan et al., 2008). It is worth mentioning that bone as a load-bearing organ has been shaped with excellent mechanical properties to bear mechanical loads induced by daily human activities. Thus, mechanical stimulus represents a crucial factor affecting the PLA degradation, and how the mechanical properties of PLA vary during the degradation of PLA scaffolds should be well revealed to understand the mechanically regulated bone recovery process. The influence of the mechanical stimulus on the PLA scaffold has already been studied in literatures (Fan et al., 2008; Li et al., 2017). However, most literature treated the effect of the mechanical stimulus on the degradation of bulk PLA materials instead of porous PLA scaffolds. Moreover, although many mathematical models have been proposed to describe the PLA degradation (Gopferich, 1996; Han and Pan, 2009; Chen et al., 2011; Sackett and Narasimhan, 2011; Shi et al., 2018), few studies verified the models by designing corresponding *in vitro* experiments and quantitively described the relationship between the mechanical stimulus and the PLA scaffold degradation.

To this end, we performed an *in vitro* 90-day degradation experiment to investigate the effect of the mechanical stimulus on the degradation of a 3D-printed porous scaffold. Correspondingly, a mathematical model was developed to describe the scaffold degradation as well. To imitate the *in vivo* environment, the scaffolds were subjected to two intermittent mechanical stimuli in the phosphate buffer saline (PBS) on the basis of the loading intensity during daily human activities. The mass, volume fraction, Young’s modulus, and number average molecular weight of the PLA scaffolds were measured during the entire *in vitro* degradation experiment. Moreover, the experimental and simulation results of the Young’s modulus and normalized number average molecular weight were compared. It might be stated that the mechanical stimulus–induced degradation framework of the porous scaffolds could be beneficial to the BTE scaffold design.

## *In vitro* Experiment

### Scaffold Preparation

**FIGURE 1:**
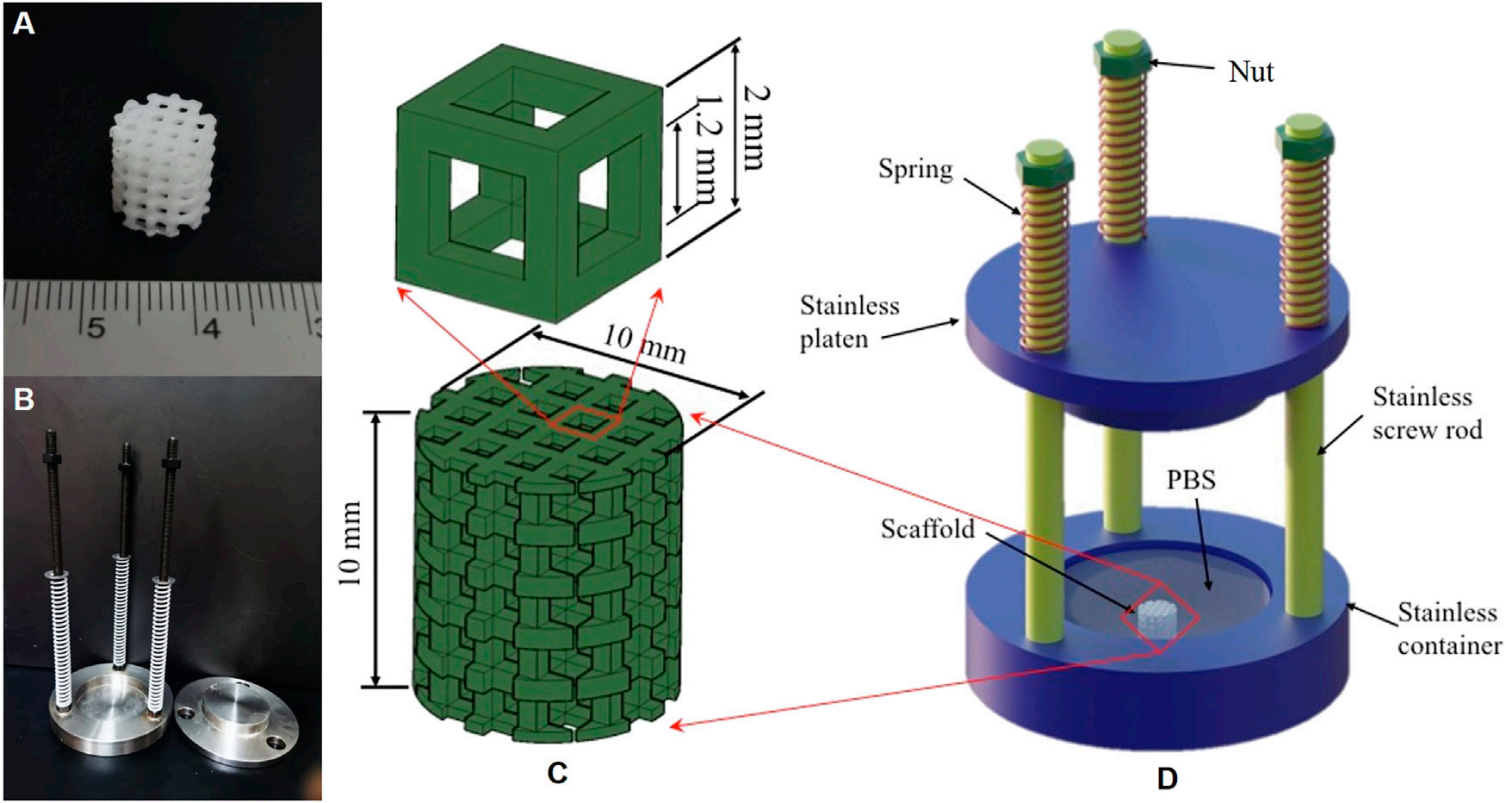
The photo of a 3D-printed scaffold **(A)**; the loading device used in the *in vitro* compressive experiment **(B)**; the topological scaffold and its RVE **(C)**; and diagram of the loading device **(D)**.

The raw PLA filaments with diameter 1.75 ± 0.02 mm were purchased from a chemical enterprise (Huiwei, China). A fused deposition modeling–based 3D-printer with resolution 0.01 mm (Raise3D Pro2, China) was used to fabricate 45 PLA scaffolds with diameter and height 10 mm (see Figure 1A). The scaffolds shared a periodic structure with porosity 64.8%. The designed topological structure with a representative volume element (RVE) (Shui et al., 2019) is shown in Figure 1C.

### Experimental Methods

In order to study the effect of the mechanical stimulus on the degradation of the 3D-printed PLA scaffolds, a loading device was custom-designed, as shown in Figure 1B. The device consisted of four parts: three springs, three screw rods with nuts, an upper plate for loading, and a bottom container, see Figure 1D. Adjusting the spring compression could control the mechanical stimulus level. In detail, the spring stiffness was first calibrated using a material testing machine (Instron 5943 Inc., United States), and then the applied load was calculated by multiplying the spring stiffness and the distance of screwing the nut along the screw rod. The loads generated by the spring compression and the plate weight were uniformly applied to the scaffolds in the container, which was filled with phosphate buffer saline (PBS, 1×, Hyclone, GE Healthcare, United States) to mimic the *in vivo* environment.

The fabricated 45 scaffolds were divided into two groups (21 in each group, and the rest 3 were shared by the two groups), and two compressive mechanical stimuli (i.e., 0.5 and 1.0 MPa) were respectively applied to the groups. It is worth mentioning that 3 of 21 scaffolds in each group were compressed for 3 h per day for 90 days by the corresponding stimulus in the air, and this aimed to eliminate the possibility of the scaffold failure due to the pure mechanical stimuli instead of the degradation in the PBS. The shared three samples by the two groups were tested for day 0, and the other 18 scaffolds in each group were immersed in PBS and also compressed for 3 h per day for 90 days. Every 15 days, three scaffolds were taken out and tested. The grouping strategy and scaffold distribution are illustrated in Table 1. In addition, the PBS solution was replaced every 2 days to maintain the pH value of the experimental environment, and the spring compression was adjusted after each scaffold was taken-out to maintain the constant mechanical stimuli (i.e., 0.5 and 1.0 MPa) on the rest of the immersed scaffolds. Four degradation indices including mass, volume fraction, Young’s modulus, and normalized number averaged molecular weight were characterized.

**TABLE 1:** Grouping strategy and distribution of the 45 scaffolds.

Groups (MPa)	Air	PBS						
	Day 90#	Day 0	Day 15	Day 30	Day 45	Day 60	Day 75	Day 90
0.5	3	3	3	3	3	3	3	3
1.0	3		3	3	3	3	3	3

Noted: Day 90# represents that the scaffolds were compressed for 90 days in the air.

### Mass Measurement

The taken-out scaffolds were first dried in a drying oven (WGL-65B, Tianjin Taiste Instrument Co. Ltd., China) at 37°C for 48 h, and then weighed by a balance (FA1004N, Shanghai Minqiao Precision Scientific Instruments Co., Ltd., China) to determine the mass of the residual scaffolds.

### μCT-Derived Volume Fraction

Micro-CT is an effective method to characterize the morphology of 3D-printed structures (Markl et al., 2017), and it was here used to characterize the morphological change and calculate the volume fraction of the degraded scaffolds. The vivaCT 80 system (SCANCO Medical AG, Switzerland) was first employed to examine the scaffolds with the operation parameters set at 55 keV, 145 μA, and 32 mm FOV with an integration time of 200 ms. The spatial resolution was 1,200 ppi, and there were around 500–600 slices for each scaffold. Then the micro-CT slices were imported into a piece of software to reconstruct the scaffolds by setting the minimum grey threshold value to 127 HU (Hounsfield unit) and the maximum to 255 HU. On the basis of the reconstructed scaffolds, the morphological changes of the scaffolds were characterized, and the volume fractions of the degraded scaffolds were calculated. Herein, the volume fraction of the scaffolds was expressed as SV/TV, where SV is the residual PLA volume and TV is the original scaffold volume.

### Mechanical Test–Derived Young’s Modulus

After measuring the mass and calculating the volume fraction of the scaffolds, a single column mechanical testing machine (Instron 5943 Inc., United States) with loading capacity 10 kN and accuracy 0.01 N was used to test the compressive mechanical behavior of the scaffolds. All the scaffolds were compressed with quasi-static loads at the speed of 2 mm/min. The nominal stress and strain curves were recorded, which were used to calculate the Young’s modulus of the degraded scaffolds.

### Calculation of the Normalized Number Average Molecular Weight

The number average molecular weight of the scaffolds was examined by a PL-GPC220 gel permeation chromatograph system (Agilent Technologies, United States). We weighed 6 mg of the fragmented scaffolds dissolved in tetrahydrofuran (THF) to get 2 ml solution with a concentration of 3 mg/ml as a preparation. The analysis was carried out at 40°C in a PLgel 5 μm MIXED-C 300 × 7.5 mm column, and THF was used as eluent with the flow rate set as 0.1 ml/min. Then the normalized number average molecular weight was calculated by *M*_*n*_(*t*)/*M*_*n*_(0), in which *M*_*n*_(*t*) was the measured number average molecular weight at day *t* and *M*_*n*_ (0) ≈ 8 × 10^4^ g/mol was the measured number average molecular weight of the scaffold at day 0.

### Statistical Analysis

Four degradation indices were presented in the mean ± standard deviation. In order to analyze the intragroup significance between different time points and the intergroup significance between different mechanical stimuli, a one-way ANOVA test was performed on the four indices, and the *p*-value < 0.05 indicated a significant difference.

## Numerical Simulation

### Mathematical Model of PLA Degradation

A modified pseudo-first-order kinetics function was used to describe the normalized number average molecular weight *β*(*t*) of the constituent PLA [the ratio of *M*_n_(*t*) and *M*_*n*_ (0) (Shi et al., 2018)]. Under the mechanical stimulus, *β*(*t*) was expressed as:

$$\beta \left(t\right)=\frac{M_{\text{n}}\left(t\right)}{M_{\text{n}}\left(0\right)}=e^{-\lambda t},$$ (1)

with

$$\lambda =\lambda_{\sigma }\cdot \lambda_{a},$$

where *λ*_*σ*_ and *λ*_*a*_ were degradation rates representing the effects of the mechanical stimulus and autocatalysis, respectively, and *λ*_*σ*_ was a modified Arrhenius function (Zhurkov and Korsukov, 1974; Li et al., 2017) as:

$$\lambda_{\sigma }=\exp\left[a+\left(b-1/RT\right)\left(E_{0}-\alpha \sqrt[{3}]{\sigma }\right)\right],$$ (2)

where *E*_0_ was the initial activity energy of the PLA. *a*, *b*, and *α* were material-dependent parameters, σ was the magnitude of the mechanical stimulus, and *T* and *R* represented the Kelvin temperature and the molar gas constant, respectively. *λ*_*a*_ was expressed as:

$$\lambda_{a}=e^{C_{m}},$$ (3)

where *C*_*m*_ was the concentration of hydrolysates induced by the autocatalysis with *C*_*m*_ = 0 at day 0. The release-diffusion process of the hydrolysates was introduced by Fick’s second law (Chen et al., 2011) as:

$$\frac{\partial C_{m}}{\partial t}=\nabla \left(D_{0}e^{\varphi \left(1-\beta \left(t\right)\right)}\cdot \nabla C_{m}\right)+S\left(t\right),$$ (4)

where *S*(*t*) denoted the source of hydrolysates, *D*_0_ represented the initial diffusion coefficient of non-hydrolyzed constituent PLA, and *φ* is a material-dependent constant.

To judge whether a PLA scaffold element was completely degraded: on the one hand, we assumed that the element was completely degraded when *β*(*t*)*<β*_*thre*_ was satisfied. On the other hand, considering the stochastic event of the hydrolysis-induced PLA chain breakage, the degradation probability density function $p\left(t\right)=ke^{-k\lambda t}$ was applied, where *k* was a coefficient referring to the meshing density of the finite element model (Shi et al., 2018). If the degradation probability $\int_{t}^{t+dt}p\left(t\right)dt$ was less than a randomly generated number from 0 to 1, then the element was assumed to be completely degraded.

The mechanical properties of the polymer were related to its number average molecular weight. The relationship between the Young’s modulus *E*_*s*_(*t*) and normalized number average molecular weight *β*(*t*) of the constituent PLA was expressed as:

$$E_{s}\left(t\right)=E_{s}+\left(\frac{E_{s}-E_{solu}}{9}\right)\left[1-\beta^{-1}\left(t\right)\right],$$ (5)

where *E*_*solu*_ was the Young’s modulus of the PBS, which was treated as an incompressible solid.

### Numerical Implementation of the Mechanical Degradation Model

**FIGURE 2:**
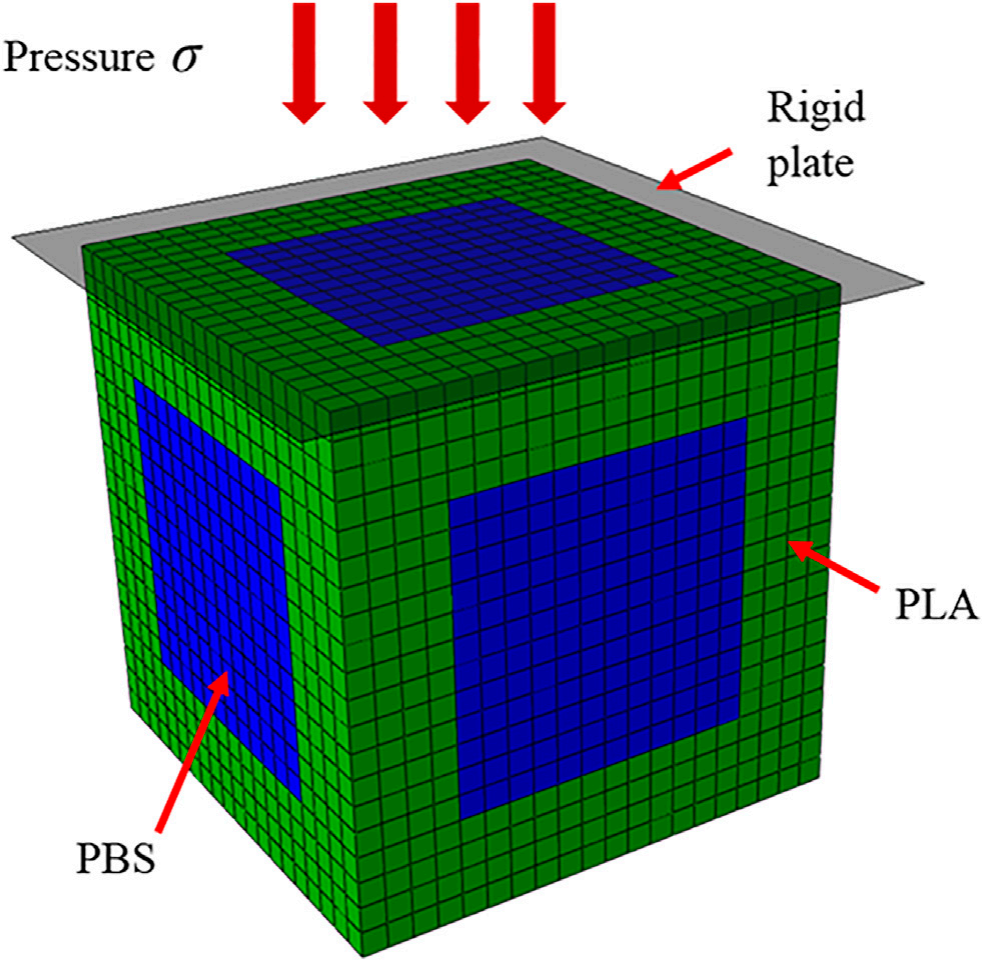
Loading diagram of the meshed RVE in the numerical simulation.

To numerically study the periodic PLA scaffold degradation, the RVE of the scaffold in Figure 1C was treated in Abaqus (DS SIMULIA, United States) without loss of generality. The RVE was meshed into 8,000 elements (Figure 2), in which the green and blue elements represented the PLA and PBS, respectively. For each PLA element, the above degradation model was coded through subroutine in Abaqus (VUMAT) to mimic the degradation behavior of the PLA scaffold.

Consistent with the two mechanical stimuli in the *in vitro* experiments, a rigid plate was placed on the top of RVE, and two pressures (0.5 and 1.0 MPa) were perpendicularly applied on the plate (see Figure 2). Moreover, 90 cycles were simulated for the 90-days degradation process, each cycle represented the 3-h mechanical stimulus per day on the scaffold model. The input parameters in the simulation were listed in Table 2. The Young’s modulus and averaged normalized number average molecular weight were compared between the simulations and the *in vitro* experiments. It is worth mentioning that in the numerical simulation, *β*(*t*) of the degraded scaffolds varied from element to element, thus we calculated the averaged *β*(*t*) by dividing the sum of *β*(*t*) of all residual PLA elements by the number of the elements.

**TABLE 2:** Input parameters for simulation.

Parameters	Symbol	Value	Unit
Degradation rate constant ratio	*λ*_0_	0.0075 (Li et al., 2017)	day^−1^
Dimension ratio	*k*	0.217	—
State change threshold	*β*_*thre*_	0.01 (Shi et al., 2018)	—
Temperature	*T*	298	K
Initial diffusion coefficient	D_0_	1.2 × 10^−9^ (Gleadall et al., 2014)	m^2^/day
Material constant for diffusion	*φ*	9.43 (Gleadall et al., 2014)	—
Young’s modulus of PLA	*Es*	400 (Middleton and Tipton, 2000)	MPa
Young’s modulus of PBS	*E*_*solu*_	1 (Shi et al., 2018)	MPa
Poisson’s ratio of PLA	*υ*_*s*_	0.3 (Shi et al., 2018)	—
Poisson’s ratio of PBS	*υ*_*solu*_	0.49 (Shi et al., 2018)	—
Initial activity energies of PLA	*E*_*0*_	79.52 (Li et al., 2017)	kJ/mol
Constant parameters	*a*	−2.16 (Li et al., 2017)	—
Constant parameters	*b*	0.354 (Li et al., 2017)	mol/kJ
Constant parameters	*α*	22 (Li et al., 2017)	kJ/(mol·MPa)
Molar gas constant	*R*	0.008314	kJ/(mol·K)

## Results and Discussion

### Morphology, Mass, and Volume Fraction

**FIGURE 3:**
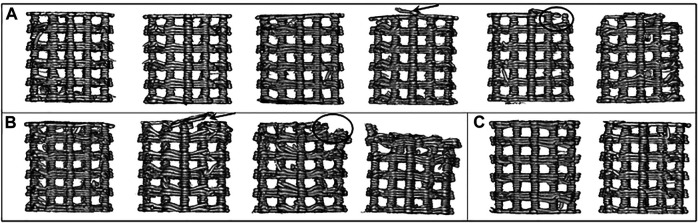
The μCT-based reconstructed scaffolds in the degradation experiment. **(A)** The degraded scaffolds in the 0.5 MPa group at day 15, 30, 45, 60, 75, and 90; **(B)** the degraded scaffolds in the 1.0 MPa group at day 15, 30, 45, and 60; and **(C)** the scaffolds compressed in the air in the 0.5 and 1.0 MPa groups.

The morphologies of the reconstructed scaffolds subjected to the two mechanical stimuli 0.5 and 1.0 MPa are shown in Figure 3. In the 0.5 MPa group, the scaffolds were reconstructed at six time points (Figure 3A), while in the 1.0 MPa group, there were only four reconstructed scaffolds at day 15, 30, 45, and 60 (Figure 3B) as scaffolds were crushed or fragmented at day 75 and 90. Moreover, the scaffolds in the two groups compressed in the air at day 90 were also reconstructed (Figure 3C). In detail, Figure 3 shows the weak morphological change of the scaffolds under the low mechanical stimulus (0.5 MPa) in the first 45 days. With the cycling stimulus, the curled bar at the top of the dried scaffold appears at day 60 (the indication of the arrow), and the top of the scaffolds starts fragmenting at day 75 (see the circled part), which attributes to the top-down loading method of *in vitro* experiments. The curl-up and fragment phenomena correspond to the morphology of the scaffolds under the high mechanical stimulus (1.0 MPa) at day 30 and 45, respectively. The morphological change illustrates that the bar curl-up of the scaffolds is the precursor of the failure, and the high mechanical stimulus accelerates the degradation process thus advances the scaffold failure. In addition, the scaffolds compressed in the air for 90 days are almost intact under the two mechanical stimuli. Compared to the failed scaffolds compressed in the PBS, this indicates that the scaffold failure indeed resulted from degradation in PBS instead of pure mechanical stimuli.

**FIGURE 4:**
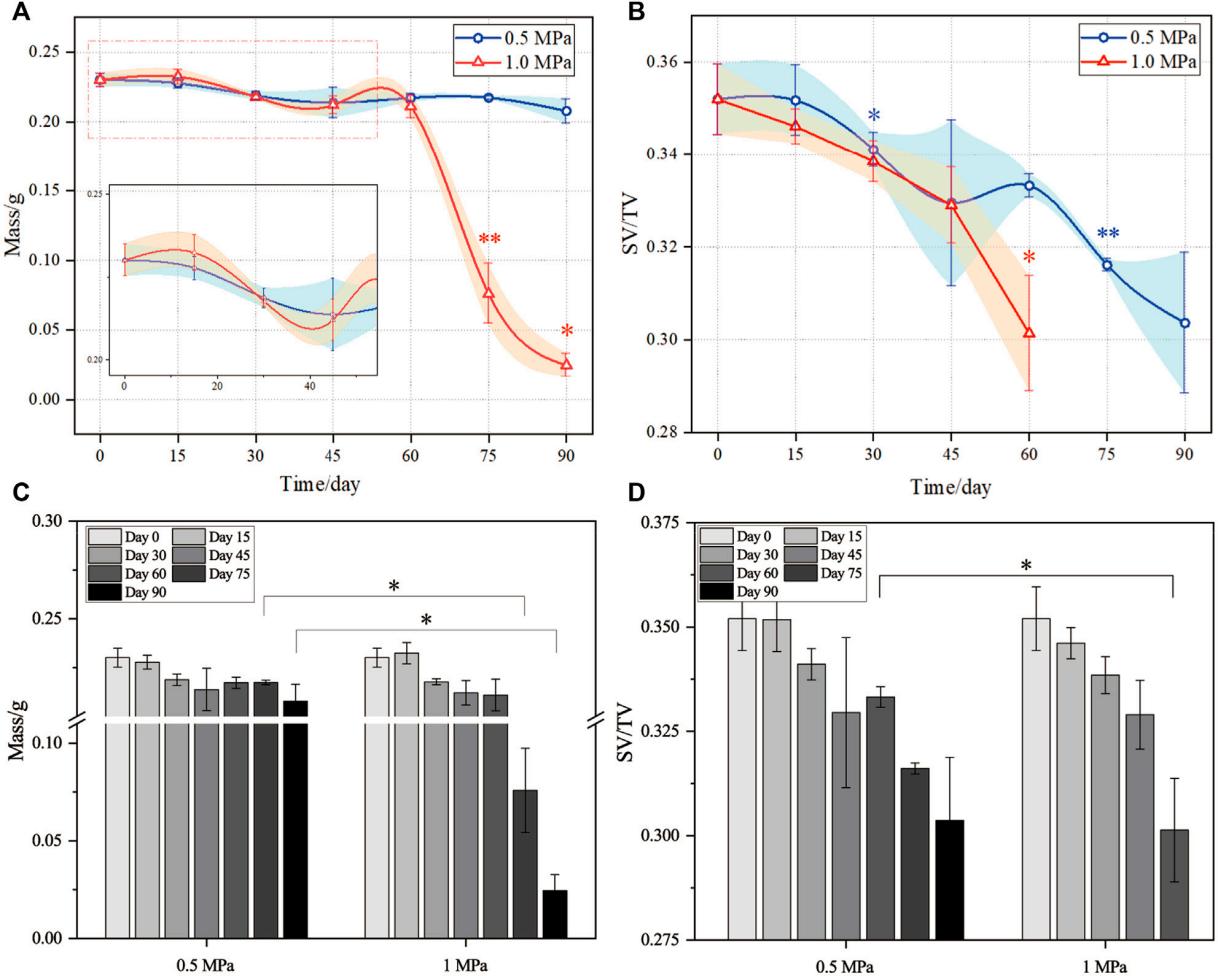
Evolutions of the mass and volume fraction in the degradation experiment. **(A)** and **(C)** mass; **(B)** and **(D)** volume fraction. Scaffold number was three for each test (*n* = 3). The error bars show standard deviation. ANOVA tests were performed for statistical analysis. *,** in **(A)** and **(B)** indicates the intragroup significant difference between the current time point with respect to its previous time point; * in **(C)** and **(D)** indicates the intergroup significant difference between the low and high mechanical stimuli. ∗ indicates *p* < 0.05, ∗∗ indicates *p* < 0.01.

The mass and volume fraction of the degraded scaffolds in the experiment are depicted in Figure 4. Generally, the mass of the scaffolds under the two mechanical stimuli fluctuates (see Figure 4A). The mass of the scaffolds varies similarly before day 60 (the insert of Figure 4A) but differently after day 60. Moreover, after day 60, the intragroup comparison of mass between different time points is significantly different in the 1.0 MPa group, which is different from that of the 0.5 MPa group. This might attribute to more fragments in the 1.0 MPa group, which increased the contact between the scaffold and PBS and led to the acceleration of the hydrolysis of the PLA. Plus, the fragmented scaffolds were subjected to high stress concentration, which also fostered the scaffold degradation. The intergroup comparison between the two mechanical stimuli was significantly different at day 75 and 90 (see Figure 4C).

The evolution of the volume fraction (SV/TV) of the scaffolds is shown in Figure 4B. Generally, the volume fraction decreases in a fluctuating way for the 0.5 MPa group but in a stable way for the 1.0 MPa group before day 60. The intragroup comparison of the volume fraction showed a significant degradation at day 30 and 75 for the 0.5 MPa group and at day 60 for the 1.0 MPa group. This might be due to the competition between the water uptake and degradation which coexisted in the degradation. In the degradation process, the water uptake changed the molecular network of the PLA and swelled the scaffold bars (Chen et al., 2007) even though the samples were dried before characterization (Gottlieb, 1988; Ayrilmis et al., 2019). Thus, volume fraction changes induced by the water uptake fluctuated the degradation curve in the 0.5 MPa group, while the faster degradation in the 1.0 MPa group offset the swelling effect induced by the water uptake. The intergroup comparison between the two mechanical stimuli was significantly different at day 60 (see Figure 4D). Anyhow, the statistical analyses of the mass and volume fraction indicates that the high mechanical stimulus accelerated the scaffold degradation at the late stage of the degradation process.

### Young’s Modulus

**FIGURE 5:**
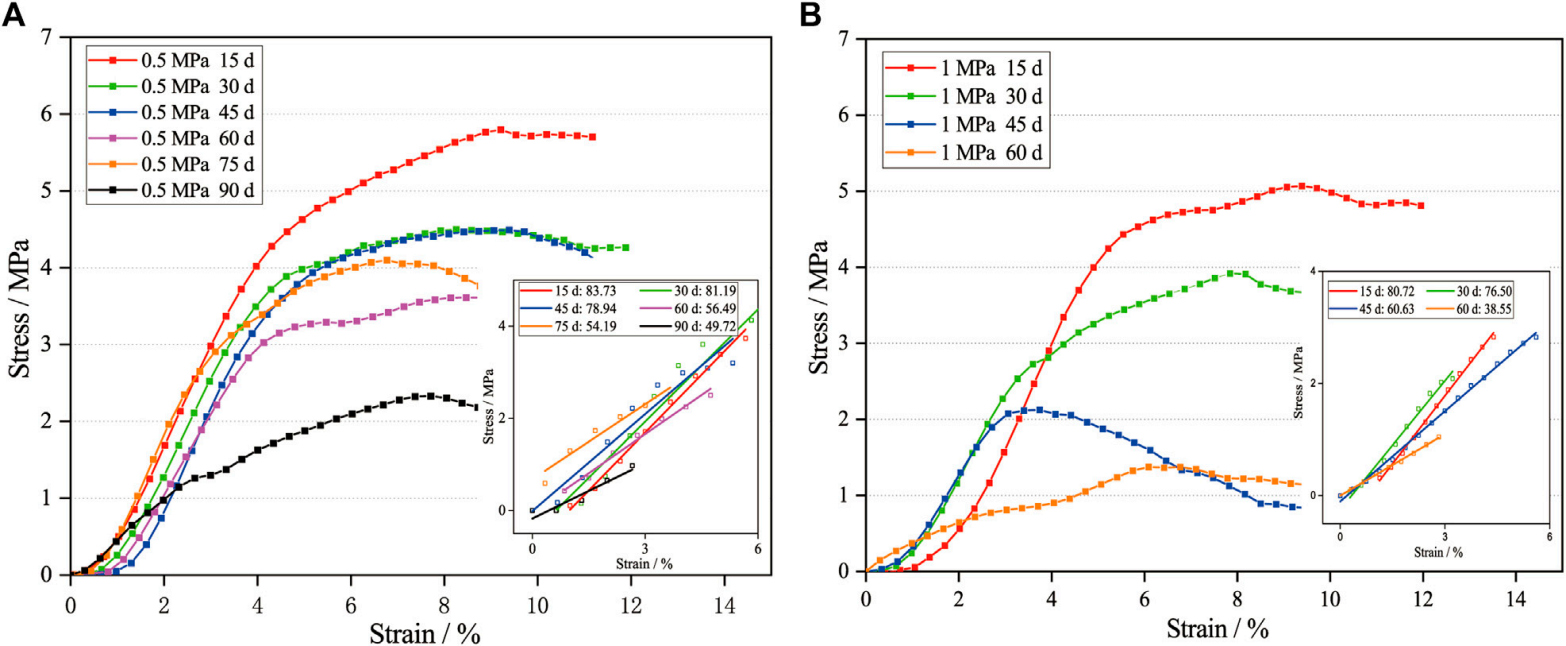
The stress and strain curves of selected degraded scaffolds in the 0.5 MPa **(A)** and 1.0 MPa **(B)** groups. Note: the Young’s modulus after time points in insets is MPa.

The dried scaffolds were compressed and their stress and strain curves were recorded by a mechanical testing machine to calculate the Young’s modulus. The stress and strain curves of the scaffolds in the two groups are plotted in Figure 5, respectively. The Young’s modulus is obtained by fitting the linear and elastic stages of the curves, see the insets. It is noted that the strengths of the scaffolds also decrease during the scaffold degradation. Namely, the strengths of the 0.5 MPa group from day 15–90 are 5.97, 3.98, 3.78, 3.12, 3.02, and 1.26 MPa, respectively, which correspond to the first inflection point of the stress and strain curves. Similarly, the strengths of the 1.0 MPa group are 4.67, 2.72, 2.07, and 0.82 MPa, respectively. The decrease of the Young’s modulus and strengh of the macroscopic scaffold attributed to the degradation of the microscopic scaffold bar (Revati et al., 2017). In detail, the intermittent mechanical stimuli are similar to the low-cycle loading on a 3D-printed PLA-based scaffold (Senatov et al., 2016), which reported more or greater micro-damages or micro-voids at the interface between the printed layers under high mechanical stimulus, and the degradation of the PLA scaffold was fostered due to the surfaces of the increased micro-damages exposed to PBS. In addition, the micro-damages or micro-voids improved the water absorption of the PLA (Singh et al., 2000; Islam et al., 2010), and at these sites, the stress concentration further reduced the mechanical properties of the PLA scaffold (Yew et al., 2005). Therefore, the high mechanical stimulus resulted in scaffold failure at the early time, as shown in Figure 3.

**FIGURE 6:**
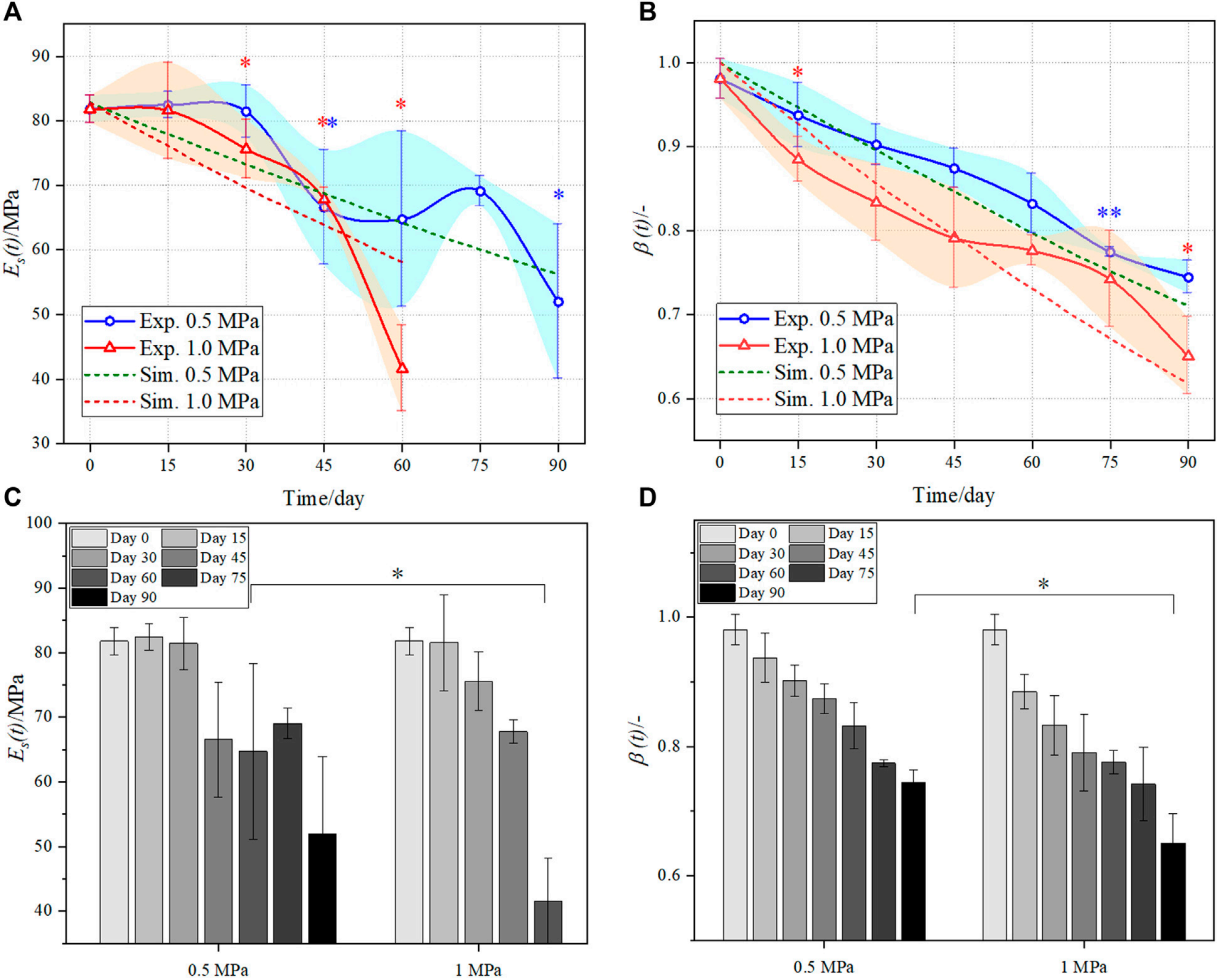
Comparison of the evolved Young’s modulus **(A)** and **(C)** and number average molecular weight **(B)** and **(D)** in the two groups between the experiment (solid line) and the simulation (dotted line). Sample number was three for each test (*n* = 3). The error bars show standard deviation. ANOVA tests were performed for statistical analysis. *,** in **(A)** and **(B)** indicates the intragroup significant difference between the current time point with respect to its previous time point; * in **(C)** and **(D)** indicates the intergroup significant difference between the low and high mechanical stimuli. * indicates *p* < 0.05, ** indicates *p* < 0.01.

The comparison of the Young’s modulus between the experiments and the numerical simulation is demonstrated in Figure 6A. The decreasing trends of the Young’s modulus in the two methods are roughly consistent, and the numerical results show that the high mechanical stimulus leads to faster loss of the scaffold’s Young’s modulus, while the experimental result is not as stable as that in the simulation. In particular, the intragroup comparison of the Young’s modulus shows a significant reduction for the 0.5 MPa group at day 45 and 90 and for the 1.0 MPa group at day 30, 45, and 60. The instability may be due to uncontrollable factors in experiments, for example, the uncontrolled micro-structures of scaffolds in the manufacturing process, and studying a single RVE instead of the whole scaffold may be another reason. Moreover, the intergroup comparison of the Young’s modulus shows that the Young’s moduli of the two groups are significantly different at day 60, see Figure 6C. This indicates the consistency with the mass and volume fraction, which highlights that the high stimulus accelerated the scaffold degradation at the late stage of the degradation process.

### Normalized Number Average Molecular Weight

The number average molecular weight *M*_n_ (*t*) is a significant parameter to characterize the degradation of the PLA scaffolds. The comparison of *β* (*t*) between the experiments and the simulation is shown in Figure 6B. Likewise, the *β* (*t*) of the scaffolds in the 1.0 MPa group generally reduces faster than the 0.5 MPa group. This can be easily obtained from Eq. 2, which shows that the greater stress reduces more initial activity energy *E*_0_ of the PLA hydrolysis, and thus accelerates the scaffold degradation. It is worth mentioning that the *β* (*t*) at day 0 is 1.0 in the simulation, while it is less than 1.0 in the experiment. This is because the scaffolds in the experiments experienced initial degradation due to environment humidity before the test (Chen et al., 2011). Different from the mass, volume fraction, and Young’s modulus, the *β* (*t*) of the two groups does not fluctuate in the degradation process and decreases more apparently at the early stage (before day 45). Furthermore, the quasi-linearity of the degradation curves was consistent with the compressed PLA samples under pressure 1.0 MPa (Li et al., 2017), in which the authors measured the viscosity average molecular weight. The intragroup comparison of the *β* (*t*) shows a significant reduction for the 0.5 MPa group at day 75 and for the 1.0 MPa group at day 15 and 90. The intergroup comparison of the *β* (*t*) shows that the *β* (*t*) of the two groups are significantly different at day 90, see Figure 6D. Again, this indicates that the high mechanical stimulus accelerates the scaffold degradation at the late stage of the degradation process.

## Conclusion

In this study, the degradation of a 3D-printed porous PLA scaffold under mechanical stimulus was studied by combining the *in vitro* experiments with the numerical simulations. Both the experiments and simulations indicated that the PLA scaffold degradation was generally accelerated due to the mechanical stimulus and a high mechanical stimulus led to faster scaffold degradation, but uncontrollable factors instabilized the degradation behavior of the scaffolds under the low mechanical stimulus. Moreover, the high mechanical stimulus strongly influenced the scaffold degradation at the late stage of the degradation (after day 60). The present study has revealed the degradation behavior of a 3D-printed PLA scaffold under the mechanical stimulus and might be helpful for the future biodegradable esters-based BTE scaffolds.

## Data Availability

The original contributions presented in the study are included in the article/Supplementary Material; further inquiries can be directed to the corresponding authors.
